# Protocol for development of a checklist and guideline for transparent reporting of cluster analyses (TRoCA)

**DOI:** 10.1136/bmjopen-2025-099609

**Published:** 2025-08-21

**Authors:** Daniil Lisik, Syed Ahmar Shah, Rani Basna, Tai Dinh, Ryan P Browne, Jeffrey L Andrews, Meredith Wallace, Absalom Ezugwu, Ana Marusic, Dat Tran, Joaquín Torres-Sospedra, Hieu-Chi Dam, Philippe Fournier-Viger, Christian Hennig, Marieke Timmerman, Matthijs J Warrens, Eva Ceulemans, Bright I Nwaru, Tina M Hernandez-Boussard

**Affiliations:** 1 Department of Public Health and Clinical Medicine, Section of Sustainable Health, The OLIN Unit, Umeå Universitet, Umeå, Sweden; 2 Krefting Research Centre, Institute of Medicine, University of Gothenburg Sahlgrenska Academy, Gothenburg, Sweden; 3 The University of Edinburgh Usher Institute, Edinburgh, UK; 4 Division of Geriatric Medicine, Department of Clinical Sciences, Lund University, Malmö, Sweden; 5 CMC University, Hanoi, Vietnam; 6 The Kyoto College of Graduate Studies for Informatics, Kyoto, Japan; 7 Department of Statistics and Actuarial Science, University of Waterloo, Waterloo, Ontario, Canada; 8 Department of Statistics, The University of British Columbia, Vancouver, British Columbia, Canada; 9 Department of Psychiatry, Statistics and Biostatistics, University of Pittsburgh, Pittsburgh, Pennsylvania, USA; 10 Unit for Data Science and Computing, North-West University, Potchefstroom, South Africa; 11 Department of Research in Biomedicine and Health, University of Split School of Medicine, Split, Croatia; 12 University of Canberra, Canberra, Australian Capital Territory, Australia; 13 Department of Computer Science, Universitat de València, Valencia, Spain; 14 Japan Advanced Institute of Science and Technology, Nomi, Japan; 15 Big Data Institute, Shenzhen University College of Computer Science and Software Engineering, Shenzhen, Guangdong, China; 16 Department of Statistical Sciences ‘Paolo Fortunati’, University of Bologna, Bologna, Italy; 17 Department of Psychometrics and Statistics, University of Groningen, Groningen, Netherlands; 18 GION Education/Research, Department of Pedagogical and Educational Sciences, University of Groningen, Groningen, The Netherlands; 19 Quantitative Psychology and Individual Differences, KU Leuven, Leuven, Belgium; 20 University of Gothenburg, Wallenberg Centre for Molecular and Translational Medicine, Gothenburg, Sweden; 21 Department of Biomedical Data Science, Stanford University, Stanford, California, USA

**Keywords:** Health informatics, Information technology, Information management

## Abstract

**Abstract:**

**Introduction:**

Cluster analysis, a machine learning-based and data-driven technique for identifying groups in data, has demonstrated its potential in a wide range of contexts. However, critical appraisal and reproducibility are often limited by insufficient reporting, ultimately hampering the interpretation and trust of key stakeholders. The present paper describes the protocol that will guide the development of a reporting guideline and checklist for studies incorporating cluster analyses—Transparent Reporting of Cluster Analyses.

**Methods and analysis:**

Following the recommended steps for developing reporting guidelines outlined by the Enhancing the QUAlity and Transparency Of health Research Network, the work will be divided into six stages. Stage 1: literature review to guide development of initial checklist. Stage 2: drafting of the initial checklist. Stage 3: internal revision of checklist. Stage 4: Delphi study in a global sample of researchers from varying fields (*n*=≈) to derive consensus regarding items in the checklist and piloting of the checklist. Stage 5: consensus meeting to consolidate checklist. Stage 6: production of statement paper and explanation and elaboration paper. Stage 7: dissemination via journals, conferences, social media and a dedicated web platform.

**Ethics and dissemination:**

Due to local regulations, the planned study is exempt from the requirement of ethical review. The findings will be disseminated through peer-reviewed publications. The checklist with explanations will also be made available freely on a dedicated web platform (troca-statement.org) and in a repository.

**Strengths and limitations of this study**

- The modified Delphi approach will contribute to a rigorous and quantifiable basis for consensus.
- Stakeholders and panellists from a broad range of fields and backgrounds will contribute with perspectives on various application contexts.
- Stringent criteria for item inclusion will ensure robust agreement.
- The wide applicability of the planned framework, while enhancing its versatility across diverse contexts, may result in a lack of specific details tailored to particular research areas.

## Introduction

 Artificial intelligence (AI), particularly machine learning (ML), a subset of AI in which algorithms learn patterns from data without being explicitly programmed,^1^ has revolutionised our capability to analyse, understand and make use of complex (multimodal) data. One of the main domains of ML is cluster analysis, which constitutes a family of data-driven techniques to identify groups within data.^2^ Cluster analysis has been used in a wide variety of contexts, from identifying subtypes of diseases^3^ to prioritising urban governance patterns^4^ and assessing water quality.^5^ Similarly, many partitioning methods are available, with the rapid development of novel and improved techniques. Clustering algorithms rely on varying assumptions about the data (eg, data types/distribution) and the concept of clustering (eg, segmenting by shape or density of data).^6^ In addition, data preprocessing approaches, clustering algorithm settings (hyperparameters) and criteria for selecting the optimal clustering solution can all heavily influence clustering outputs. For the above reasons, transparent and comprehensive reporting of computational aspects is essential for critical appraisal and comparison of results. Unfortunately, however, such reporting is often inadequate. This issue is common in most fields, including health sciences,^7^,^11^ social sciences,^12^,^14^ engineering^15^ and economics,^16 17^ ultimately hampering interpretation, trust and development pace.

Reporting guidelines—defined by the EQUATOR (Enhancing the QUAlity and Transparency Of health Research) Network as ‘checklists, flow diagrams or explicit text to guide authors in reporting a specific type of research, developed using explicit methodology’^18^—are increasingly used to improve the quality and comprehensiveness of reporting.^19^ The suggested benefits of reporting guidelines are many, for example, increasing transparency of methodological aspects and enabling a more accurate comparison between studies.^20^ Although adherence generally remains suboptimal,^21^ there is evidence indicating that reporting guidelines improve reporting,^22^,^24^ which in turn catalyses methodological development and innovation by highlighting limitations to be bridged in subsequent research.^20^ Finally, reporting guidelines may directly increase research quality,^25^ particularly if incorporated at early stages of research.^26 27^

There is an abundance of reporting guidelines for AI-based studies, most focusing on supervised learning applications, such as TRIPOD+AI^28^ for clinical prediction models. Nevertheless, to the best of our knowledge, for cluster analyses, no reporting guideline is available or registered at the EQUATOR Network as under development. Although different ML methods share implementational aspects (and consequently aspects important to report),^29^ cluster analyses are conducted in ways that fundamentally differ from studies in other ML domains (eg, generally lacking ‘ground truth’ labels),^30^ warranting a dedicated reporting guideline. The present protocol outlines the planned processes for developing a new consensus-based reporting guideline named TRoCA (Transparent Reporting of Cluster Analyses), comprising a user-friendly checklist, accompanying explanation and elaboration (E&E) and examples of good reporting of cluster analyses.

### Focus and scope of TRoCA

Given the many heterogeneous contexts in which cluster analyses are applied, we aim to develop a reporting guideline that is, as far as possible, context-agnostic. In other words, we aim to make TRoCA applicable to most studies that use cluster analysis as a data-driven technique to discover groups within data, regardless of the application. We will accomplish this primarily by focusing on quantitative aspects of clustering pertaining to data preprocessing, modelling and cluster characterisation and interpretation. To this end, we will exclude aspects that are non-computational or specific to certain research areas or data types, as guidance for handling such issues may vary substantially between fields and is oftentimes more appropriately covered by other reporting guidelines. There are several notable examples of (largely) context-agnostic reporting guidelines, for example, in systematic reviews, where the use of PRISMA^31^ (Preferred Reporting Items for Systematic Reviews and Meta-Analyses), a general reporting guideline for systematic reviews, is used in combination with more specific reporting guidelines depending on study designs^32^ or meta-analysis approaches.^33^

Although cluster analyses may incorporate varying degrees of manual labelling of groups (eg, semisupervised learning, in which a subset of the data is labelled while groups are inferred by the algorithm for the remaining data),^2^ TRoCA will be applicable for *unsupervised* cluster analyses, that is, segmentation of cases without using any available labelling. This delimitation was primarily done to streamline the reporting guidelines and checklist; however, given that most clustering applications are unsupervised, we still expect our work will have broad applicability. For semisupervised cluster analyses, and for specific studies where elements in TRoCA are lacking or redundant/irrelevant, researchers may modify the checklist according to their needs.

## Methods and analysis

**Figure 1:**
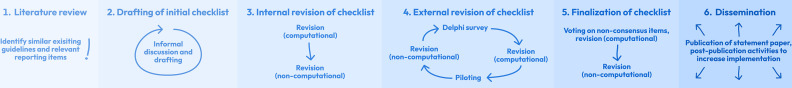
Overview of the structure of the planned study.

The overall process of developing the reporting guideline will largely follow the recommendations outlined by the EQUATOR Network. Deviations from these recommendations will mainly pertain to costly and logistically challenging functions, such as face-to-face consensus meetings, which will be substituted with online correspondence and meetings, and postpublication activities, including translation of the guidelines, which we do not see as necessary at this time.^18^ Furthermore, we have drawn inspiration from methodological reviews of reporting guidelines.^1934^,^37^ As mentioned in the EQUATOR Network recommendations,^18^ there is no single best approach for conducting this type of study. The structure of the study will be partly based on the protocol^38^ outlined for the development of TRIPOD+AI (a reporting guideline for ML-based diagnostic/prognostic prediction model studies), given the somewhat overlapping technological context and aims. Some compromises (primarily regarding the physical meetings and organisational size) will be made due to time and funding constraints. The project has been registered as under development on the EQUATOR Network. The statement manuscript will be drafted in accordance with the ACcurate COnsensus Reporting Document reporting guidelines.^39^ Each stage of the planned process is detailed below and summarised graphically in figure 1. Stage 1 is expected to commence in August 2025 and the completion of Stage 5 (ie, finalisation of manuscript(s)) by the end of January 2026.

### Stage 1: literature review

A literature review will be performed with two main objectives: (1) an in-depth assessment of the current state of reporting in the cluster analyses literature, including areas of particular need for improvement and overlapping/differing reporting items; and (2) to gather reference material from similar reporting guidelines/checklists. Two databases (Google Scholar and PubMed) will be searched. Google Scholar has been chosen as it indexes the full texts of articles in a broad range of non-medical journals, and PubMed has been chosen due to a broad indexing of medical journals, with health sciences constituting the largest proportion of scientific literature.^40^ Although not providing a complete overview of academic literature, these two databases have largely satisfactory coverage.^41^ From each database, papers from the past 10 years will be assessed. For objective (1), the following search queries will be constructed (syntax modified according to the database): (‘review’ OR ‘methodology’ OR ‘quality’ OR ‘transparency’) AND ‘cluster analysis’. For objective (2), similarly: (‘guideline’ OR ‘checklist’) AND ‘cluster analysis’. Finally, the EQUATOR Network will be screened for related guidelines.

### Stage 2: drafting and internal revision of checklist

Based on the findings from the literature review and on domain expertise, an initial checklist will be drafted. This will be carried out through an informal collaborative process in a live-synchronised Microsoft Word (Microsoft Corp., USA) document by the *computational core group* (n=4), who were identified and invited by DL without strict formal inclusion/exclusion criteria, based on their substantial experience in statistics, ML and, in particular, cluster analysis. The goal with the initial checklist is to construct a comprehensive set of reporting items with short explanations, divided into sections (eg, data preprocessing) for ease of use and interpretation. This initial draft checklist will be revised in two rounds:

- *Round 1 (revision based on computational aspects)*. This will be performed by informal input through the aforementioned live-synchronised Microsoft Word document from the *internal collaborators*, a group of researchers (n=20) with varying backgrounds and experience in incorporating cluster analyses as part of their research (working in 15 countries across five continents, in fields ranging from population genetics to geoinformatics), who were identified and invited by DL without strict formal inclusion/exclusion criteria. The size and composition of the *internal collaborators* group were chosen such that it balances (broad) representation and administrative load. The *internal collaborators* will be able to suggest the addition/removal/rephrasing of reporting items and sections, focusing on ensuring comprehensive coverage of elements of importance for the critical appraisal and reproducibility of cluster analyses. DL will collate these suggestions, and the *computational core group* will finalise the revision. Efforts will be made to exclude clearly irrelevant items to maximise response rates in the subsequent Delphi study.^42^
- *Round 2 (revision based on non-computational aspects)*. This will be performed within the live-synchronised Microsoft Word document by informal input from a *non-computational core group* (n=4), who were identified and invited by DL without strict formal inclusion/exclusion criteria, based on their substantial experience in meta-research, development of reporting guidelines and evidence synthesis. The *non-computational core group* will provide suggestions and collectively discuss the addition/removal/rephrasing of reporting items and sections, focusing on the applicability and usability of the form, ultimately aiming at making the checklist flexible and enhancing adherence, ease of use and interpretation for reviewers and readers.

### Stage 3: external revision of checklist

**Figure 2:**
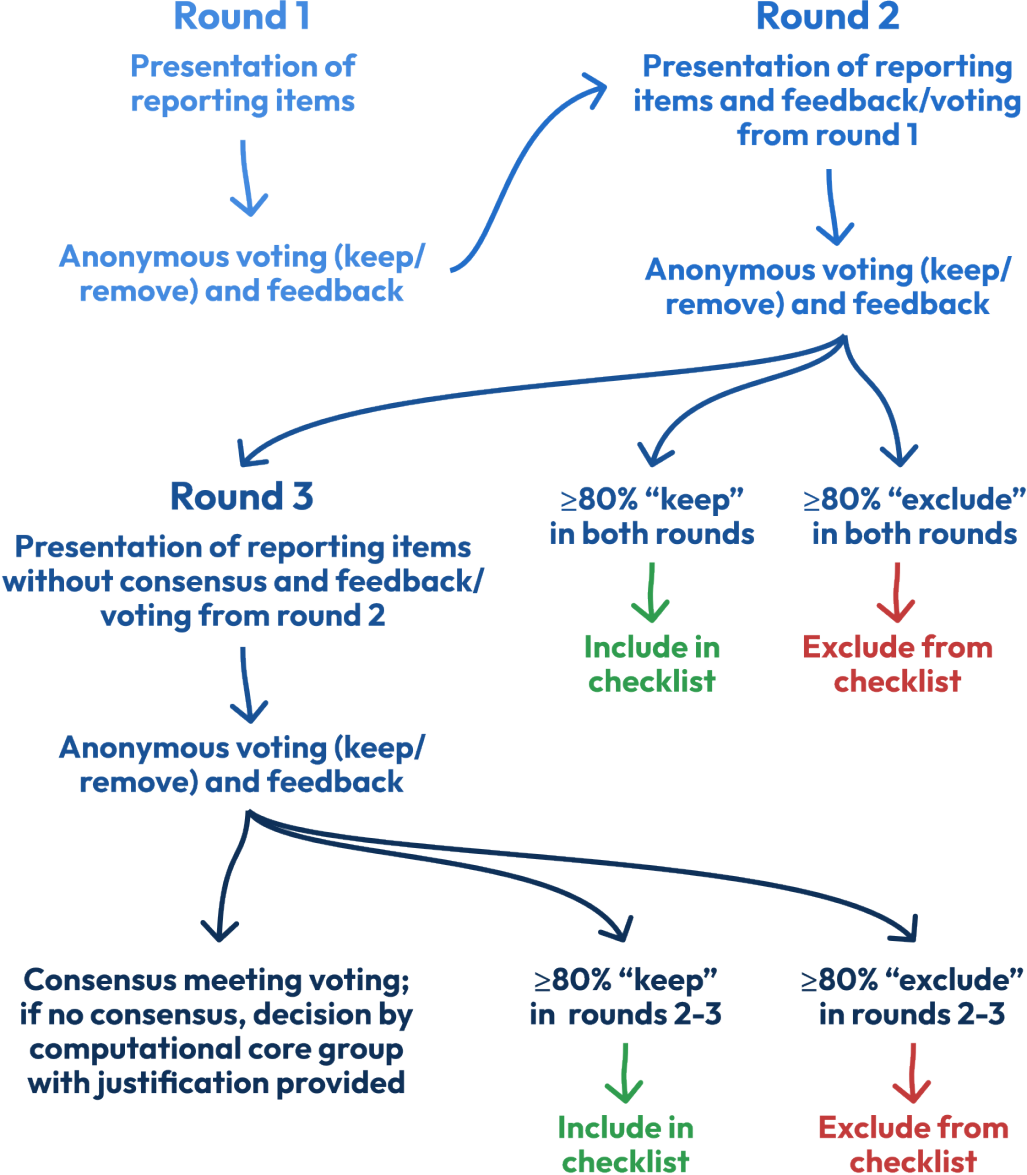
Flow diagram of the modified Delphi consultation process to arrive at consensus regarding the items to include/exclude from the reporting checklist from the perspective of reporting items being introduced, voted on and subsequently kept or excluded. Each round is separated by collation of feedback, revision based on feedback, piloting, etc.

At this stage, the initial checklist will be revised further through a modified Delphi consultation process as a formal method for attaining consensus for the finalisation of the checklist items.^19 39^ The benefit of a Delphi method over singular researchers providing recommendations stems from the ‘wisdom of crowds’ theory^43^ by providing a structured, quantifiable, anonymous and iterative process of summarising opinions (and ultimately attaining consensus) of a panel of experts.^44^ There is no well-established definition of the construction of Delphi processes, nor of what constitutes a ‘modified’ Delphi process,^34^ but it generally includes an iterative (for a (predefined) set of rounds) process of anonymous (thus less likely to be prone to dominance of individual experts or group conformity) rating/feedback from panellists and a structured summary of previous rounds’ rating/feedback, allowing for panellists to change their mind given the arguments and data of the collective group. Statistical aggregation of various kinds is used to assess whether consensus has been achieved.^35 45^ Specifications of the planned Delphi process will be largely based on the recommendations in the methodological literature and administrative/logistical compromises. A visual summary of the Delphi process to be used is shown in figure 2.

A wide range in the number of panellists has been used in previous Delphi-based studies,^20 35 37^ with a simulation study suggesting that as few as ~20 panellists can suffice to yield reliable results if using similarly trained experts in a well-defined domain^46^ or homogeneous stakeholders. In more complex and heterogeneous multistakeholder contexts, 60–80 panellists have been suggested to achieve a high level of replicability, with relatively modest improvements beyond these levels but nevertheless with additional benefits such as improved validity and potentially increased subsequent implementation.^44^ For the present work, given the many domains of utilisation in cluster analysis, we aim to recruit 160 panellists for the first Delphi round, which, estimating up to a 40% attrition rate based on previous similar studies,^28 47^ would leave an acceptable ~60 panellists by round three. Based on the acceptance rate in contact attempts for the *internal collaborators* (~50%) and previous similar studies,^28 47^ a comparable or even lower acceptance rate is expected for the *external collaborators*, who will form the panel in the Delphi process. Thus, ~300 researchers (or more until 160 panellists are achieved) will be invited. To ensure a diverse representation of domains/fields, five roughly equal groups (~50 researchers each) of these will be selected based on their predominant work within the natural sciences, education, engineering, social sciences and economics. From health sciences, ~80 researchers will be contacted, to reflect that a large proportion of studies using cluster analysis are undertaken within clinical research (and roughly representing the proportion of health science publications in the academic literature).^40^ Beyond this, the following criteria will be used for selecting panellists in an admittedly arbitrary but systematic and relatively reproducible fashion (it should also be noted that the somewhat relaxed criteria may lead to a more heterogeneous and therefore more informative panel)^37^: (1) ≥3 peer-reviewed published works from the past 5 years in which cluster analysis was used, (2) h-index≥10 or total number of citations≥500, (3) listed in a peer-reviewed published work, as per above, as having contributed to statistical analysis (or the like) or is known by DL or other co-authors to be experienced in cluster analysis.

Researchers will be identified through broad searches on relevant databases (Google Scholar and PubMed), screening editorial board/reviewer pages of relevant journals (to ensure representation of journal editors) and through professional networks of the *computational core group, non-computational core group* and *internal collaborators*, with subsequent confirmation of suitability by the two former groups. The researchers will be invited by DL through a personalised email, containing information about the purpose and structure of the study, the right to withdraw from the study and have any submitted data deleted at any time without needing to provide a reason, the researcher’s role and recognition as a panellist (name and affiliation in supplementary material and on the public website of the project), as well as a link to an online consent form. The researcher will be informed that the aim of the checklist is to provide ease of use and inclusion of the minimum set of reporting items needed from the vast majority/all cluster analysis studies to sufficiently facilitate reproducibility of the cluster analysis and critical assessment of quality and risk of bias (and thereby comparison with other studies, either through uptake in subsequent studies or structured synthesis through systematic reviews or meta-analyses).

Each researcher who has provided informed consent to participate in the Delphi study (panellist) will then be invited to each round, regardless of whether he/she participated in the previous round. This approach is superior in maintaining representation of the baseline panel and minimising the risk of false consensus.^36^ The Delphi study will be performed using Microsoft Forms (Microsoft Inc., USA) to efficiently distribute and evaluate the surveys. The form will contain the name and description for each reporting item, along with a checkbox through which the panellist can vote to keep or remove the reporting item through a checkbox. For each reporting item, there will also be a voluntary free-text box to provide justification for the rating and a voluntary free-text box to suggest improvement in the phrasing. At the end of each section, an additional voluntary free-text box will be available to provide suggestions for additional reporting items (this will only be available in the first round to ensure that each reporting item can be assessed at least two times if needed). Finally, at the end of the form in round one, the panellist will be able to rate one or two non-computational aspects regarding visual/structural details (eg, presence of colours in the table, wording of column headings, layout, etc) of the reporting checklist in a similar binary fashion. We believe that the binary rating system, in contrast with rating scale approaches, will provide a more implicit/genuine view^48^ and improve response rates.^49 50^ Three weeks will be given to complete the form in each round, with one reminder email sent to non-responders 1 week before each deadline and another reminder email 1 day before the deadline, as well as a maximum of 1 week between rounds to decrease attrition rates.^51^ Efforts will be made to increase interest and sense of ownership in the study results, reminding the panellists of their fundamental impact on the checklist, as this also may help reduce attrition rates.^52^ Ratings and feedback from incomplete forms will be considered in the same way as fully completed forms.

The Delphi process will be performed in a maximum of three rounds, a commonly chosen upper limit that has several benefits, eg, minimised risk of panellist fatigue (which may increase risk of false consensus).^35 53^ In the first round, the initial checklist (from Stage 2) will be evaluated by the panellists. After the first round is completed, the ratings and feedback will be collated and discussed within the *computational core group* to revise wordings. Additional reporting items suggested by panellists will be added if at least 50% (2/4) of the *computational core group* approves them (internal informal voting in a Microsoft Word document). Furthermore, the checklist will be piloted by students/colleagues (*n*=3, arbitrarily defined) of members of the *computational core group*, who will provide additional feedback on usability (time allocated, difficulties/ambiguities, etc). Finally, the *non-computational core group* will also be consulted for suggestions for improvement in an informal manner with the aforementioned input/revision in context (eg, relating to wording). In the second round, the initial checklist (revised as per above) and added items (as per above) will be evaluated by the panellists. In this round, there will not be a free-text box for suggesting new reporting items (to ensure that each reporting item can be evaluated at least two times). Additionally, for each reporting item, the percentage for each rating decision will be displayed, together with anonymised comments justifying the two rating decisions as well as the panellist’s previous rating, if such is available. In all other aspects, round two will be performed as in the first round, with the same postround procedures (except for no assessment of suggestions for new reporting items). At round three, only items which have not yet attained consensus from the panellists will be evaluated, while those attaining such status will only be displayed for information purposes (although a free-text box to further polish wording will remain). Consensus has been defined in many ways in Delphi studies, although a percentage of agreement is the most commonplace, with 75% being the median in a systematic review of such studies (range: 50%–97%), all levels ultimately being arbitrary.^35^ Given that binary rating measures may provide higher rates of consensus due to the mere format,^50^ we will define consensus as 80% of panellists rating either ‘keep’ or ‘remove’. Beyond this, some argue that ‘stability’ (eg, ≤15% change in responses across two rounds)^54^ should be an additional criterion.^34^ Given that feedback indicating a high level of agreement triggers an increase in agreement and vice versa,^55^ it is not obvious how stability should be defined or interpreted as a consensus criterion. For this reason, we will use a concept of ‘stability beyond threshold’, which means that to attain the status of consensus, a reporting item must have received agreement of ≥80% for two rounds (for inclusion) or ≤20% for two rounds (for exclusion). If all reporting items have attained consensus after round two, round three will not be performed. Reporting items that do not attain consensus after round three will be evaluated at the consensus meeting (Stage 4).

### Stage 4: consensus meeting to finalise checklist

The *computational core group*, the *non-computational core group* and the *internal collaborators* will meet in a virtual consensus meeting to finalise the checklist. Prior to this meeting, DL will collate all rating data and feedback provided thus far and send it out to participants 1 week in advance. Reporting items that have attained consensus in the modified Delphi consultation process will automatically be included in the checklist, although specific wordings may still be altered slightly at this stage. Reporting items that have not attained consensus will be voted on, and if failing to reach 80% (for inclusion) or 20% (for exclusion), the *computational core group* will informally make the final decision with explicit justification.^35^ The *non-computational core group* will have the final decision regarding the non-computational elements (wording, layout, etc). The dissemination strategy will also be informally discussed. All rating/feedback data, together with notes and voting data from the consensus meeting (for which one participant in the meeting will be selected to be responsible for collating), will be made available on an online repository (link in Ethics and dissemination). If substantial changes are made, an additional pilot (performed as per Stage 3) will be performed to provide comparison data on usability.

### Stage 5: production of manuscript(s)

The *computational core group* and *non-computational core group* will prepare a draft of the statement manuscript. The *internal collaborators* will start participating at the revision stage, during which all of the aforementioned co-authors will provide edits and comments for suggestions. Depending on the length of the manuscript and the checklist, an additional manuscript (E&E), as recommended by the EQUATOR Network,^18^ will be produced to provide in-depth rationale for inclusion and good examples of each reporting item.

### Stage 6: dissemination

We aim to publish in at least one high-impact journal, and possibly multiple key journals (depending on practical/logistical factors), to ensure broad outreach to different readership groups. The publications will be open access to further increase implementation, and the checklist will be made available in a repository and a dedicated web platform. We will also seek indexing on the EQUATOR Network and contact relevant journals to recommend the use of TRoCA in submission guidelines. The reporting guidelines will be further disseminated through relevant conferences, social media and courses by the authors. Additional postpublication activities recommended by the EQUATOR Network, such as evaluating impact, developing extensions and adherence assessment forms,^18^ will also be evaluated for relevance.

## Discussion

TRoCA is anticipated to widely benefit the field of cluster analysis by providing a broad consensus-based foundation for reporting such studies, subsequently increasing transparency and improving the precision and clarity with which similar works may be compared. We believe that TRoCA will be useful in most clustering contexts due to its context-agnostic and minimalistic composition, which may be modified or complemented with other reporting guidelines if needed for highly specialised studies. Finally, researchers will be able to guide the development of cluster analysis-based studies at early stages to proactively make more informed and sensible methodological decisions.

### Ethics and dissemination

The research described in this protocol is exempt from the requirement of ethical review according to Swedish regulations (Ethical Review Act), which is further explained in an official document from the University of Gothenburg (see online supplemental file 1). All Delphi study participants will be asked for their informed consent before taking part, and the Delphi studies will be performed in accordance with the Helsinki Declaration. The findings of this study will be disseminated through peer-reviewed publications. The final checklist and detailed explanation for each reporting item will also be made freely available in a repository^56^ and on a dedicated web platform (troca-statement.org).

## References

[1] Sarker IH (2021). Machine Learning: Algorithms, Real-World Applications and Research Directions. SN Comput Sci.

[2] Hennig C, Meila M (2015). Handbook of Cluster Analysis.

[3] Ye L, Pien GW, Ratcliffe SJ (2014). The different clinical faces of obstructive sleep apnoea: a cluster analysis. Eur Respir J.

[4] Mokhles S, Davidson K, Acuto M (2024). Unveiling urban governance diversity: Clustering cities based on mitigation actions. Ambio.

[5] Du X, Shao F, Wu S (2017). Water quality assessment with hierarchical cluster analysis based on Mahalanobis distance. Environ Monit Assess.

[6] Frades I, Matthiesen R, Matthiesen R (2010). Bioinformatics Methods in Clinical Research.

[7] Rivera E, Corte C, DeVon HA (2020). A systematic review of illness representation clusters in chronic conditions. Res Nurs Health.

[8] Busija L, Lim K, Szoeke C (2019). Do replicable profiles of multimorbidity exist? Systematic review and synthesis. Eur J Epidemiol.

[9] Horne E, Tibble H, Sheikh A (2020). Challenges of Clustering Multimodal Clinical Data: Review of Applications in Asthma Subtyping. JMIR Med Inform.

[10] van Rooden SM, Heiser WJ, Kok JN (2010). The identification of Parkinson’s disease subtypes using cluster analysis: a systematic review. Mov Disord.

[11] Mello GT de, Bertuol C, Minatto G (2023). A systematic review of the clustering and correlates of physical activity and sedentary behavior among boys and girls. BMC Public Health.

[12] Bauer GR, Mahendran M, Walwyn C (2022). Latent variable and clustering methods in intersectionality research: systematic review of methods applications. Soc Psychiatry Psychiatr Epidemiol.

[13] Alexander EF, Johnson MD (2023). On categorizing intimate partner violence: A systematic review of exploratory clustering and classification studies. J Fam Psychol.

[14] Crowther D, Kim S, Lee J (2021). Methodological Synthesis of Cluster Analysis in Second Language Research. Lang Learn.

[15] Balijepally V, Mangalaraj G, Iyengar K (2011). Are We Wielding this Hammer Correctly? A Reflective Review of the Application of Cluster Analysis in Information Systems Research. JAIS.

[16] Dolnicar S (2002). A review of unquestioned standards in using cluster analysis for data-driven market segmentation. Journal of Travel & Tourism Marketing.

[17] Crum M, Nelson T, de Borst J (2022). The use of cluster analysis in entrepreneurship research: Review of past research and future directions. Journal of Small Business Management.

[18] Moher D, Schulz KF, Simera I (2010). Guidance for developers of health research reporting guidelines. PLoS Med.

[19] Schlussel MM, Sharp MK, de Beyer JA (2023). Reporting guidelines used varying methodology to develop recommendations. J Clin Epidemiol.

[20] Simera I, Moher D, Hoey J (2009). The EQUATOR Network and reporting guidelines: Helping to achieve high standards in reporting health research studies. Maturitas.

[21] Jin Y, Sanger N, Shams I (2018). Does the medical literature remain inadequately described despite having reporting guidelines for 21 years? - A systematic review of reviews: an update. J Multidiscip Healthc.

[22] Moher D (2018). Reporting guidelines: doing better for readers. BMC Med.

[23] Tan ZW, Tan AC, Li T (2020). Has the reporting quality of published randomised controlled trial protocols improved since the SPIRIT statement? A methodological study. BMJ Open.

[24] Han S, Olonisakin TF, Pribis JP (2017). A checklist is associated with increased quality of reporting preclinical biomedical research: A systematic review. PLoS ONE.

[25] Panic N, Leoncini E, de Belvis G (2013). Evaluation of the endorsement of the preferred reporting items for systematic reviews and meta-analysis (PRISMA) statement on the quality of published systematic review and meta-analyses. PLoS ONE.

[26] Moher D, Shamseer L, Clarke M (2015). Preferred reporting items for systematic review and meta-analysis protocols (PRISMA-P) 2015 statement. Syst Rev.

[27] Marušić A (2015). A tool to make reporting checklists work. BMC Med.

[28] Collins GS, Moons KGM, Dhiman P (2024). TRIPOD+AI statement: updated guidance for reporting clinical prediction models that use regression or machine learning methods. BMJ.

[29] Kapoor S, Cantrell EM, Peng K (2024). REFORMS: Consensus-based Recommendations for Machine-learning-based Science. Sci Adv.

[30] Choi RY, Coyner AS, Kalpathy-Cramer J (2020). Introduction to Machine Learning, Neural Networks, and Deep Learning. Transl Vis Sci Technol.

[31] Page MJ, McKenzie JE, Bossuyt PM (2021). The PRISMA 2020 statement: an updated guideline for reporting systematic reviews. BMJ.

[32] Stubbs JL, Thornton AE, Sevick JM (2020). Traumatic brain injury in homeless and marginally housed individuals: a systematic review and meta-analysis. Lancet Public Health.

[33] Giacoppo D, Laudani C, Occhipinti G (2024). Coronary Angiography, Intravascular Ultrasound, and Optical Coherence Tomography for Guiding of Percutaneous Coronary Intervention: A Systematic Review and Network Meta-Analysis. Circulation.

[34] Nasa P, Jain R, Juneja D (2021). Delphi methodology in healthcare research: How to decide its appropriateness. World J Methodol.

[35] Diamond IR, Grant RC, Feldman BM (2014). Defining consensus: a systematic review recommends methodologic criteria for reporting of Delphi studies. J Clin Epidemiol.

[36] Boel A, Navarro-Compán V, Landewé R (2021). Two different invitation approaches for consecutive rounds of a Delphi survey led to comparable final outcome. J Clin Epidemiol.

[37] Shang Z (2023). Use of Delphi in health sciences research: A narrative review. Medicine (Baltimore).

[38] Collins GS, Dhiman P, Andaur Navarro CL (2021). Protocol for development of a reporting guideline (TRIPOD-AI) and risk of bias tool (PROBAST-AI) for diagnostic and prognostic prediction model studies based on artificial intelligence. BMJ Open.

[39] Gattrell WT, Logullo P, van Zuuren EJ (2024). ACCORD (ACcurate COnsensus Reporting Document): A reporting guideline for consensus methods in biomedicine developed via a modified Delphi. PLoS Med.

[40] Publications Output: U.S Trends and international comparisons. https://ncses.nsf.gov/pubs/nsb20214/publication-output-by-country-region-or-economy-and-scientific-field.

[41] Bramer WM, Rethlefsen ML, Kleijnen J (2017). Optimal database combinations for literature searches in systematic reviews: a prospective exploratory study. Syst Rev.

[42] Gargon E, Crew R, Burnside G (2019). Higher number of items associated with significantly lower response rates in COS Delphi surveys. J Clin Epidemiol.

[43] Surowiecki J (2005). The Wisdom of Crowds.

[44] Manyara AM, Purvis A, Ciani O (2024). Sample size in multistakeholder Delphi surveys: at what minimum sample size do replicability of results stabilize?. J Clin Epidemiol.

[45] Rowe G, Wright G (1999). The Delphi technique as a forecasting tool: issues and analysis. Int J Forecast.

[46] Akins RB, Tolson H, Cole BR (2005). Stability of response characteristics of a Delphi panel: application of bootstrap data expansion. BMC Med Res Methodol.

[47] Rivera SC, Liu X, Chan A-W (2020). Guidelines for clinical trial protocols for interventions involving artificial intelligence: the SPIRIT-AI Extension. BMJ.

[48] Harvey C (2016). Binary Choice vs Ratings Scales: A behavioural science perspective. International Journal of Market Research.

[49] Sparling EI, Sen S (2011). Rating: How difficult is it?. RecSys.

[50] Rivera-Garrido N, Ramos-Sosa MP, Accerenzi M Continuous and binary sets of responses differ in the field. Sci Rep.

[51] Trevelyan EG, Robinson PN (2015). Delphi methodology in health research: how to do it?. Eur J Integr Med.

[52] Keeney S, Hasson F, McKenna H (2006). Consulting the oracle: ten lessons from using the Delphi technique in nursing research. J Adv Nurs.

[53] Humphrey-Murto S, Varpio L, Gonsalves C (2017). Using consensus group methods such as Delphi and Nominal Group in medical education research. Med Teach.

[54] Scheibe M, Skutsch M, Schofer J (2002). The Delphi Method: Techniques and Applications.

[55] Barrios M, Guilera G, Nuño L (2021). Consensus in the delphi method: What makes a decision change?. Technol Forecast Soc Change.

[56] Lisik D, Shah SA, Basna R (2024). TRoCA (Transparent Reporting of Cluster Analyses).

