# Supplementary material for: Protocol for development of a checklist and guideline for transparent reporting of cluster analyses (TRoCA)
Source: BMJ Open. 2025 Aug 21;15(8):e099609. doi: 10.1136/bmjopen-2025-099609 (PMC12374645; doi:10.1136/bmjopen-2025-099609)
Supplement: Supplementary file 1 [file bmjopen-2025-099609.reviewer_comments.pdf]

PEER REVIEW HISTORY

BMJ Open publishes all reviews undertaken for accepted manuscripts. Reviewers are asked to complete a checklist review form and are provided with free text boxes to elaborate on their assessment. These free text comments are reproduced below.

ARTICLE DETAILS

Title (Provisional)

Protocol for development of a checklist and guideline for transparent reporting of cluster analyses (TRoCA)

Authors

Lisik, Daniil; Shah, Syed Ahmar; Basna, Rani; Dinh, Tai; Browne, Ryan P; Andrews, Jeffrey L; Wallace, Meredith; Ezugwu, Absalom; Marusic, Ana; Tran, Dat; Torres-Sospedra, Joaquín; Dam, Hieu-Chi; Fournier-Viger, Philippe; Hennig, Christian; Timmerman, Marieke; Warrens, Matthijs J.; Ceulemans, Eva; Nwaru, Bright I; Hernandez-Boussard, Tina M.

VERSION 1 - REVIEW

**Reviewer**	**1**
**Name**	**Freeman, Alex**
**Affiliation**	**University College Hospital London, Department of Histopathology**
**Date**	**19-Feb-2025**
**COI**	**None**

Well written paper with clearly explained objectives. Methodology appears sound and references are provided.

**Reviewer**	**2**
**Name**	**Markham, Sarah**
**Affiliation**	**King's College, London, Biostatistics**
**Date**	**30-Jul-2025**
**COI**	**None**

This protocol concerns the much needed development of reporting guidelines for cluster analyses.
